# Role and mechanism of actin-related protein 2/3 complex signaling in cancer invasion and metastasis: A review

**DOI:** 10.1097/MD.0000000000033158

**Published:** 2022-04-07

**Authors:** Sihan Zheng, Fengfeng Qin, Ji Yin, Daiying Li, Yanlin Huang, Lanxin Hu, Lu He, Caifeng Lv, Xiaohui Li, Sen Li, Wenjian Hu

**Affiliations:** a Department of Otorhinolaryngology, The Affiliated Traditional Chinese Medicine Hospital of Southwest Medical University, Luzhou, China; b Spinal Surgery Department, the Affiliated Traditional Chinese Medicine Hospital of Southwest Medical University, Luzhou, China.

**Keywords:** Arp2/3 complex, cancer, cortactin, invasion, NPFs

## Abstract

The actin 2/3 complex (Arp2/3) regulates actin polymerization and nucleation of actin filaments, is associated with cell motility, and has been shown to play a key role in the invasion and migration of cancer cells. nucleation-promoting factor (NPF) such as N-WASP (neural-WASP famly verprolin-homologous protein family), WAVE (WASP famly verprolin-homologous protein family), and WASH (WASP and Scar homologue) undergo conformational changes upon receipt of multiple upstream signals including Rho family GTPases, *cdc42* (Cell division control protein 42 homolog), and phosphatidylinositol 4,5-bisphosphate (PtdIns 4,5 P2) to bind and activate the Arp2/3 complex. Once activated, the Arp2/3 complex forms actin-based membrane protrusions necessary for cancer cells to acquire an invasive phenotype. Therefore, how to influence the invasion and migration of cancer cells by regulating the activity of the Arp2/3 complex has attracted great research interest in recent years. Several studies have explored the effects of phosphorylation modifications of cortactin and several NPFs (Nucleation Promoting Factor) including N-WASP and WAVE on the activity of the Arp2/3 complex and ultimately on cancer cell invasiveness, and have attempted to suggest new strategies for antiinvasive therapy as a result. Other studies have highlighted the potential of targeting genes encoding partial or complete proteins of the Arp2/3 complex as a therapeutic strategy to prevent cancer cell invasion and metastasis. This article reviews the role of the Arp2/3 complex in the development, invasion, and metastasis of different types of cancer and the mechanisms regulating the activity of the Arp2/3 complex.

## 1. Introduction

Cancer cell invasion and migration are key components of cancer metastasis, which refer to the ability of cancer cells to move and cross the basement membrane and extracellular matrix, eventually moving and infiltrating into the surrounding tissue.^[1]^ Cancer invasion and metastasis are complex processes that involve multiple cellular interactions and signaling pathways. However, cancer cells must undergo changes in actin cytoskeleton regulation and produce protrusion structures on their membranes, such as lamellipodia, invadopodia, and filopodia, in order to acquire a migratory phenotype.^[2]^ The cytoskeleton is mainly composed of microfilaments, microtubules, and intermediate fibers, of which microfilaments are the main component of the cytoskeleton and are assembled by actin monomers. The actin-related protein 2/3 (Arp2/3) complex regulates the cytoskeleton, which is composed of microfilaments assembled by actin monomers. In addition to changes in the actin cytoskeleton, the formation of cellular pseudopods is required for cancer cell invasion and migration. They have been shown to consist of protrusions assembled by actin that adhere to the cell membrane and facilitates the advancement of cell, and the initial step in the migration and invasion of cancer cells is the extension of the protrusions in the direction of cell movement.^[3]^ In reality, Arp2/3 complex-mediated actin polymerization plays an irreplaceable role in the development of both plate-like and invasive pseudopods.

During the quest for a ligand for the actin-binding protein profilin, the Arp2/3 complex was discovered in Acanthamoeba castellanii.^[4]^ The Arp2/3 complex mediates actin polymerization and filament nucleation to generate branching actin networks, but it can only do so when certain NPFs (Nucleation Promoting Factor) activate it.^[5]^ In human cells, NPFs that play a role in activating the Arp2/3 complex mainly include Cortactin, the WASP (Wiskott-Aldrich syndrome protein),WAVE (WASP family verprolin-homologous protein family),WASH (WASP and Scar homologue), WHAMM (WASP homolog associated with actin, membranes and microtubules) families and JMY (junctionmediating and regulatory protein, p53 cofactor). In NPFs, WASP, WAVE, and WASH all have a VCA region at the C-terminus, which is made up of a viologen homologous structural domain (V, also known as WASP homolog 2 (WH2)), a cofilin structural domain (C, also known as the central structural domain), and an acidic structural domain (A).^[4,6,7]^ In contrast, WHAMM and JMY contain specific VCA domains. The VCA region activates the Arp2/3 complex and causes actin polymerization by binding actin monomers and activating the Arp2/3 complex. However, some phosphorylation events occurring in NPFs affect the ability of the VCA domain to bind and activate the Arp2/3 complex, which in turn regulates the formation of cellular pseudopods and the invasion and migration of cancer cells.^[6,7]^ In conclusion, the Arp2/3 complex has lately become demanding issue in cancer invasion and metastasis because it is necessary in a number of biological processes, including podocyte and platelet cell production, and offers invasive and migratory qualities to cells.

## 2. The Arp2/3 complex

### 2.1. Structure of the Arp2/3 complex

**Figure 1.**
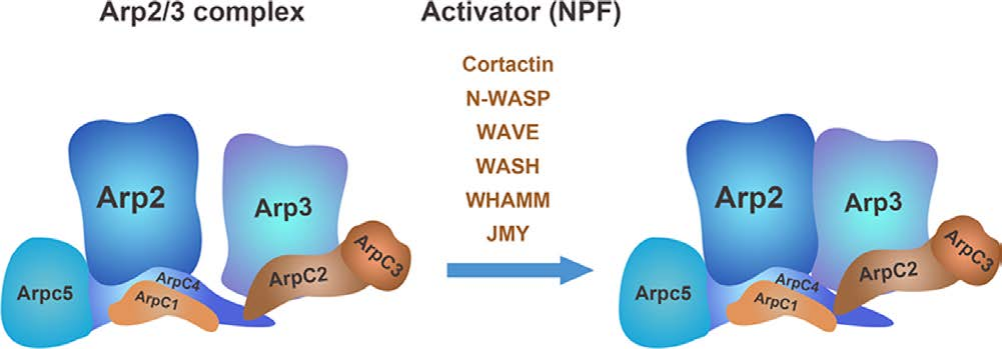
Two different conformations of the Arp2/3 complex: On the left, the Arp2/3 complex is in an inactive condition, with Arp2 and Arp3 separated by a large distance.^[5]^ When NPFs like N-WASP, WAVE, and WASH activate the Arp2/3 complex, it experiences conformational changes in which the Arp2 and Arp3 subunits approach each other and finally become the active Arp2/3 complex on the right. Arp2/3 = actin-related protein 2/3, JMY = junctionmediating and regulatory protein, NPF = Nucleation Promoting Factor, N-WASP = neural-WASP famly verprolin-homologous protein family, WASH = WASP and Scar homologue, WAVE = WASP famly verprolin-homologous protein family, WHAMM = WASP homolog associated with actin, membranes and microtubules.

Arp2/3 is a flat ellipsoid about 15 nm long, 14 nm wide, and 7 to 10 nm thick, which is currently considered to be an essential regulator of actin polymerization and actin nucleation.^[5]^ The structure of the Arp2/3 complex is shown in Figure 1. The Arp2/3 complex consists of seven subunits, of which Arp2 and Arp3 are actin-related proteins and structurally related to actin, while the other five subunits are named ARPC1, ARPC2, ARPC3, ARPC4, and ARPC5 in order of molecular weight.^[4]^ Arp2 and Arp3 fold like actin to form the first two subunits of the daughter filament. ARPC1 is a 7-bladed β-helix structure, which is currently thought to contain an embedding site and only slightly contacts actin filaments, and also binds to NPF.^[8]^ The dimers of ARPC2 and ARPC4 form a “C”-shaped pincer structure and wrap around the ARP2 and ARP3 subunits to form the structural backbone of the Arp2/3 complex and provide the main surface for the interaction of the complex with the parent filament.^[9,10]^ ARPC3 is a globular alpha helix subunit and forms a bridge between Arp3 and the parent actin filament, increasing the efficiency of nucleation.^[4]^ In addition, ARPC5 and the structurally similar ARPC3 are placed at the edge of the complex and play a key role in maintaining the structure of the complex.^[11]^ Several subunits of the Arp2/3 complex (i.e., Arp3, ARPC1, and ARPC5) have been shown to have more than one isomer, but the functional significance of these variants has not been fully investigated.^[4]^

### 2.2. The Arp/3 complex triggers actin network formation

The Arp2/3 complex plays an important role in the regulation of actin nucleation and actin filament polymerization, but the exact mechanisms of regulation have not been fully elucidated. Two models have been proposed for this regulation: dendritic nucleation and the “barbed endbranching” model. More commonly accepted today is the dendritic nucleation model, in which the Arp2/3 complex binds to one side of the mother actin filament, producing a new actin filament that branches at a 70° angle from that side, forming a Y-shaped branching network.^[12]^.

## 3. Activation of the Arp2/3 complex

The Arp2/3 complex itself is an inefficient actin nucleating agent and needs to be combined with NPF to effectively promote actin nucleation. There are NPF families in the human genome: WASP, WAVE, WASH, WHAMM, and JMY. Except for cortactin, all of these NPFs have a WCA domain (W: WASP-homology 2 domain or, WH2, C: Connector, and A: Acid) that binds to and activates the Arp2/3 complex, which is responsible for actin polymerization and nucleation.

### 3.1. Cortactin activates the Arp2/3 complex

**Figure 2.**
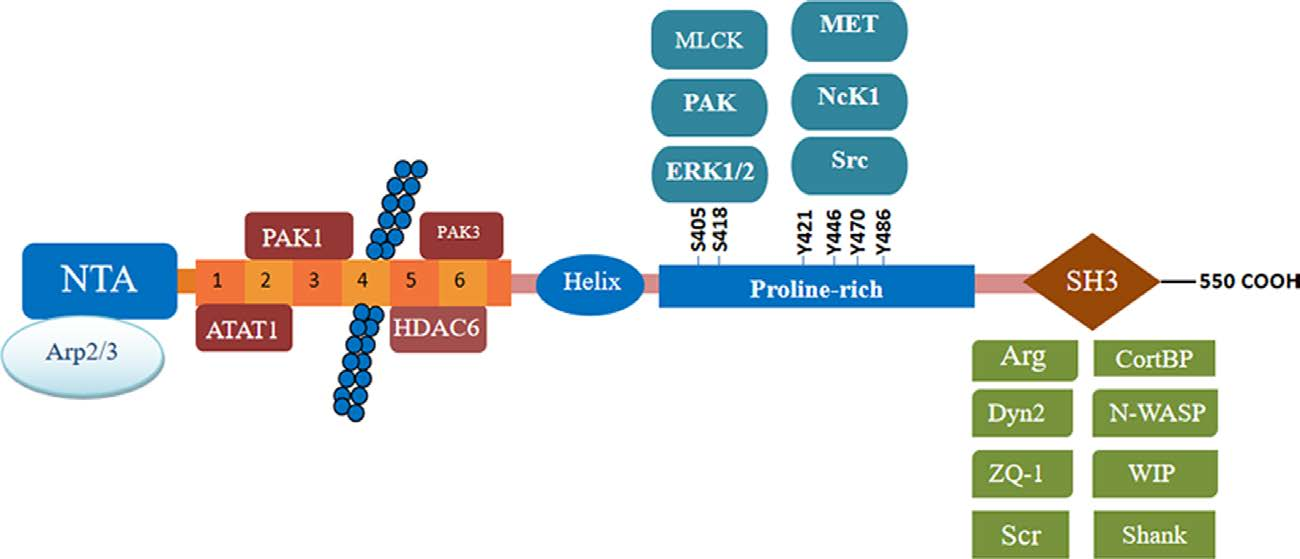
① Cortactin’s NTA binds to and activates the Arp2/3 complex, which regulates branching actin assembly as well as F-actin polymerization and contraction. ② The 6.5 F-actin repeat domain: The fourth in the repeat sequence is responsible for binding to F-actin (F-actin). Post-translational modifications of the 6.5 F-actin repeat domain can regulate the function of cortactin, and these modifications include phosphorylation and acetylation of PAK1, PAK3, ATAT1, and HDAC6. ③ An α-helix and a proline-rich region: Multiple tyrosine phosphorylation sites, such as Y421, Y446, Y470, Y486, etc., are found in these areas, which are phosphorylated by kinases such as Src, Fer, c-Met, and NCK1. ERK, PAK, MLCK, and other kinases phosphorylate two serine phosphorylation sites, S405 and S418, respectively. ④ SH3: Many cytoskeletal, membrane transport, and signaling proteins, such as N-WASP, ZO-1, CortB, and Dynamin2, bind to the C-terminal SH3 structural domain. Arp2/3 = actin-related protein 2/3, N-WASP = neural-WASP famly verprolin-homologous protein family.

The CTTN (Src substrate cortactin) gene encodes cortactin, which is found on chromosome 11q13. Many cancers, including HNSCC, oral squamous cell carcinoma, lung squamous cell carcinoma, gliosarcoma, breast cancer, colorectal cancer, and melanoma, are linked to amplification of the 11q13 segment on chromosome 11 (which includes the CTTN gene) and cortactin overexpression.^[13]^ Cortactin is a filamentous (F) actin-binding and actin 2/3 (Arp2/3) complex-activating protein with an N-terminal acidic region (NTA) that binds and activates the Arp2/3 complex. The NTA region contains a brief motif known as DDW that is necessary to bind the Arp3 component of the Arp2/3 complex, followed by six and a half repeating sequences of 37 amino acids, the fourth of which is responsible for binding to F-actin (F-actin).^[14]^ The above description of the structure of cortactin is better presented in Figure 2. Through the binding of the amino-terminal acidic domain to the Arp2/3 complex and the binding of the repeat region to F-actin, cortactin directly triggers the Arp2/3 complex’s actin nucleation activity. In addition, the SH3 structural domain of cortactin can facilitate the derepression of neural-WASP famly verprolin-homologous protein family (N-WASP) and its activation of the Arp2/3 complex by binding to the proline-rich structural domain of N-WASP.^[15]^

### 3.2. Binding and activation of the Arp2/3 complex by the VCA domain in N-WASP and WAVE

WASP protein is an actin cytoskeleton regulatory protein that is structurally divided into two types: WASP itself and N-WASP.^[16]^ The WASP protein is expressed primarily in hematopoietic cells, while its homolog, the neural (N-) WASP, is widely expressed. Including the WASP homology domain, the GTPase binding domain/Cdc42 and Rac interaction binding (GBD/CRIB) domains, and the proline-rich region, there are several functional domains shared by N-WASP and WASP.^[17]^ The VCA structural domain, the verprolin homology region (V), also known as the WASP homology 2 structural domain (WH2), the cofilin region (C), also known as the central structural domain, and the acidic region comprise the carboxyl terminus of N-WASP (A). The VCA structural domain is required for N-WASP to govern actin polymerization, with the V structural domain serving as a G-actin binding site and the CA structural domain serving as an Arp2/3 binding site. N-WASP exists in an inactive state under resting conditions and interacts via amino- and carboxy-terminal *Cdc42/Rac* interaction (also known as GBD) structural domains to mask the VCA region, thereby inhibiting the binding of the Arp2/3 complex to the CA region.^[17]^ Upstream signaling molecules such as *Cdc42* and phosphatidylinositol 4,5-bisphosphate (*PtdIns 4,5 P2*) bind and activate N-WASP, causing a conformational change in the connection between the carboxy- and amino-terminals and dissociation, eventually exposing the VCA region. We also mentioned above that the binding of N-WASP to the cortactin SH3 structural domain promotes the exposure of its own VCA structural domain and activates the Arp2/3 complex.

The WASP family verproline-homologous protein (WAVE) is divided into three types: WAVE1, WAVE2, and WAVE3. WAVE2 has been found to be broadly expressed in mammals, whereas WAVE1 and WAVE3 are found mostly in the brain but are also found throughout the mammalian body.^[18]^ WAVE is usually associated with HSPC30, NCK-associated protein 1 (*NckAP1/Hem1*), Abl interactor 1/2/3 (*Abi1/2/3*), and specifically Rac1-associated protein 1 (*Sra1/PIR121*) to form a stable WAVE protein pentamer, called the WAVE Regulatory Complex (WRC).^[19]^ In the WAVE regulatory complex (WRC), the WCA domain of WAVE is the output module of the entire complex, where the W region is the actin-binding structural domain and the C and A regions bind and activate the Arp2/3 complex.^[19]^ However, as the meander domain of WAVE interacts with Sra1 to mask the WCA domain, the WRC complex is left in a self-inhibited state in the resting state.^[20]^ Thus, the exposed activation of the WCA domain is a central part of WRC regulation. In response to extracellular stimuli, upstream signals such as Rac GTPase, phospholipids, and kinases regulate the localization and activation of WRC. The Rho-family GTPase Rac1 is the main activator of WRC, which can bind to two distinct Rac1 binding sites on the Sra1 subunit and then activate WRC by heterologous release of the WCA structural domain.^[19]^

### 3.3. Other type I NPFs: WASH, WHAMM, and JMY

In mammalian cells, WASH recruits and activates Arp2/3-mediated actin polymerization at endosomal and lysosomal membranes, which is critical for controlling endosome and vesicle morphology, fracture and various endosomal pathways, ultimately leading to degradation, recycling and retrograde transport.^[21–23]^ WHAMM is located in the endoplasmic reticulum (ER)-Golgi intermediate compartment and, similar to N-WASP, contains a WWCA motif at its C-terminus that activates Arp2/3 complex-dependent actin polymerization, but unlike N-WASP, WHAMM is not in an autoinhibited state.^[6]^ In addition to its involvement in actin assembly, WHAMM regulates vesicle transport, controls membrane shape and dynamics, and even participates in autophagy and establishes a link between Arp2/3 complex-mediated actin assembly and autophagy.^[6,24]^ Unlike other NPFs, JMY can bind and activate the Arp2/3 complex through the WCA structural domain, which contains three WH2 structural domains (WWWCA), which is involved in Arp2/3 complex-mediated protrusion and cell motility, but the exact role and mechanism are not fully understood.^[6,25]^

## 4. How activated Arp2/3 complex regulate cancer invasion and migration?

The invasion and migration of the tumor are the primary causes of poor prognosis and outcome in tumor patients, but this process also necessitates the formation of a unique structure, the cellular pseudopod, by the tumor cells themselves. Cellular pseudopods are protrusions formed by the polymerization of actin on the plasma membrane and are capable of facilitating the forward movement of cells, including lamellipodia, invadopodia, and filopodia.^[2,12]^ The lamellipodia are broad, short protrusions on the cell surface, with internal actin filaments forming a flat 2D branching network.^[12]^ The Arp2/3 complex is localized to the lamellipodia and regulates the prominence of the regulator pseudopod. For example, according to Steffen et al,^[26]^ the production of lamellipodia was greatly decreased when the expression of the ARP3 gene was down-regulated by RNA interference to lower the ARP2/3 complex in various cell types. Invadopodia are a type of actin-rich cell protrusion that protrudes ventrally from the plasma membrane and has invasive, sticky, and matrix-degrading properties, as well as being the point of convergence for several signals that regulate tumor cell invasion and migration.^[27]^ Yamaguchi et al^[28]^ used RNA interference and other techniques to demonstrate that the Arp2/3 complex is essential for invasive pseudopod production in a study on the process of invasive pseudopod generation. In conclusion, the Arp2/3 complex plays a crucial role in the formation of lamellipodia and invadopodia.

The cytoskeleton plays an important role in the invasive metastasis of tumors. At every stage of tumor cell migration, dynamic changes in the cytoskeleton occur, and the structures that undergo these dynamic changes are mostly microfilaments and microtubules. And the fact that the constriction of microfilaments at the cell tail and the lengthening of microfilaments generated by actin polymerization at the cell front are the main drivers of cell migration. Microfilaments are formed by the polymerization of actin, which in turn is mainly regulated by the Arp2/3 complex. In conclusion, the Arp2/3 complex is closely associated with dynamic changes in the cytoskeleton and the migration of tumor cells.

## 5. Impact of some phosphorylation events on the role of the Arp2/3 complex in promoting invasion and migration of associated cancers

Extracellular signals, such as growth factors, cytokines, and adhesion states, can be delivered to cortactin, N-WASP, and WAVE and regulate the activity of the Arp2/3 complex and ultimately the promotion of cancer cell invasion by the Arp2/3 complex through a variety of kinase phosphorylation mechanisms.

### 5.1. Effect of phosphorylation modification of cortactin on the activity of the Arp2/3 complex

The phosphorylation modifications of cortactin are closely related to the many functions that cortactin performs in the cell. Cortactin is phosphorylated in response to multiple stimuli, including growth factors, platelet activation, bacterial invasion, transformation, and integrin-mediated adhesion. The currently known phosphorylation sites of cortactin include mainly tyrosine and serine/threonine phosphorylation sites. The MS analysis revealed three sites of phosphorylation flanking the conserved DDW sequence in the NTA structural domain, including S11, T13, and T24, and the proximity of three sites of phosphorylation to the DDW motif suggests that phosphorylation of S11, T14, and/or T24 plays a regulatory role in the binding of cortactin to the Arp2/3 complex.^[29]^ For example, T24 phosphorylation may negatively regulate the binding and activation of cortactin to the Arp2/3 complex through spatial and electrostatic disruption mechanisms.^[30]^

### 5.2. Phosphorylation events occurring at N-WASP

The VCA structural domain of N-WASP binds to and activates the Arp2/3 complex to promote actin polymerization, but this process is regulated by multiple phosphorylation events. Dual-specificity tyrosine-phosphorylation-regulated kinase 1A (*Dyrk1A*) is reported to directly phosphorylate the GTPase binding domain (GBD) of N-WASP at three sites (Thr196, Thr202, and Thr259). Phosphorylation of GBD by *Dyrk1A* promotes interactivity with the Cdc42/Rac interactive binding (also termed GBD) domain of both the amino terminus and the carboxy terminus, and in turn inhibits Arp2/3 complex-mediated actin polymerization.^[17,31]^ According to a study, Casein kinase II binds and phosphorylates N-WASP at two sites (ser 480 and ser 481), thereby reducing the ability of the VCA structural domain of N-WASP to bind and activate the Arp2/3 complex.^[32]^ Interestingly, another study suggests that casein kinase II phosphorylates WASP Ser483/Ser484, thereby increasing the ability of WASP to activate Arp2/3 and promote actin polymerization.^[33]^ The reason for the different conclusions of the above two studies may be due to the structural differences between N-WASP and WASP. The p21-activated kinase 4 (*PAK4*) interacts directly with the N-WASP VCA structural domain and phosphorylates N-WASP at the phosphosite Ser484/Ser485 of the VCA structural domain, which concomitantly promotes Arp2/3-dependent actin polymerization in vitro. The above observation that *PAK4* contributes to Arp2/3 complex-dependent actin polymerization is consistent with the observation that phosphorylation of the VCA domain increases its affinity for the Arp2/3 complex in the study by Cory et al.^[33]^ In addition to the large number of protein kinases that phosphorylate N-WASP, there are many other protein regulators. In rectal cancer, LIM and SH3 protein 1 (*LASP1*) interact with N-WASP to activate the Arp2/3 complex, which subsequently promotes actin polymerization and facilitates colorectal cancer invasion/metastasis.^[34]^ In conclusion, since phosphorylation of N-WASP by multiple kinases can regulate the activity of the Arp2/3 complex, targeting these phosphorylation events may lead to the development of new anti-invasive therapeutic options for cancer.

### 5.3. Phosphorylation modifications of the WAVE regulatory complex

There are multiple phosphorylation sites on WAVE and Abi in WRC, which in turn are targets for a variety of kinases, including *Src, Abl*, and cyclin-dependent kinase 5 (*Cdk5*). Thus, kinases also play an important role in the activation of WRC. AbI, a non-receptor tyrosine kinase, phosphorylates Y150, which is located downstream of the Abi-1-binding fragment within the WAVE2 WHD (Tyr 151 in WAVE1 and WAVE3), interfering with the interaction between the WAVE meander region and SRA1, exposing the WCA region to Arp2/3 complex reactions and promoting Arp2/3 complex activation.^[35]^ The Src non-receptor tyrosine kinase phosphorylates Tyr125 of WAVE, similar to *AbI*, by interfering with the interaction between the meander region and sra1, thus exposing the interaction of the WCA domain with the Arp2/3 complex.^[20]^ In addition to tyrosine phosphorylation, there is phosphorylation of the serine/threonine site on WAVE. The cyclin-dependent kinases *CDK5* bind directly to WAVE2 and phosphorylate Ser-137 within the SH domain to inhibit WAVE2 activity.^[36]^ The cyclin-dependent kinases 1 (*CDK1*) phosphorylate ABI1 at serine 216, thereby attenuating ABI1-stimulated WAVE2 tyrosine phosphorylation and leading to inhibition of frontier F-actin assembly.^[37]^ *ERK* is a serine/threonine protein kinase that phosphorylates WAVE2 at the Ser343, Thr346, and Ser351 sites, thereby contributing to Rac-induced exposure of the WCA domain and activation of WRC.^[38]^ The Casein Kinase 2 (*CK2*) phosphorylates Ser482, 484, 488, 489, and 497 in the VCA region of WAVE, and this multiple phosphorylation is necessary for high affinity binding to the Arp2/3 complex.^[39]^ *PKA* (Protein kinase A) is also a serine/threonine kinase that removes inhibitory *CDK5* phosphorylation from WAVE1 by activating protein phosphatase 2A (*PP2A*).^[40]^ Thus, phosphorylation events of WAVE caused by various kinases may provide promising therapeutic strategies for the anti-invasive/metastatic nature of cancer.

### 5.4. Phosphorylation modification of the Arp2/3 complex itself

The Polo family serine threonine kinase (*Plk4*) expression is increased in a variety of cancers, including: breast, colorectal, prostate, and pancreatic cancers, and promotes cancer cell invasion and migration. It has been suggested as a therapeutic target for advanced cancers. *Plk4* phosphorylates Arp2 at the T237/T238 site, disrupting the inactive state of the Arp2/3 complex and repositioning the Arp2/3 complex to a state that allows full activation by nucleation-promoting factor (NPF).^[41]^ In another study, *NIK*, a Ste20/MAP4K4 serine/threonine kinase, led to enhanced actin nucleation activity by phosphorylating Arp2 at T237 and T238 and increasing the activity of the Arp2/3 complex.^[42]^ Phosphorylation events that occur in the Arp2/3 complex regulate its activity, but further studies are needed to determine this.

## 6. Role of the Arp2/3 complex in cancer

The Arp2/3 complex has low tissue specificity, and it has been found to be overexpressed in a variety of cancers. Regulation of the actin cytoskeleton by the Arp2/3 complex is now supposed to be the mechanism controlling tumor cell migration, invasion, and metastasis and is closely related to tumor prognosis.^[43]^ The following section presents the most relevant literature on the role of the Arp2/3 complex in various cancers and summarizes the relationships in Table 1.

**Table 1:** Arp2/3 complex subunits highly expressed in some cancers.

Name	Significantly high expression of subunits	Inhibition or silencing of the Arp2/3 complex or highly expressed subunits affects cancer invasion and migration	Related research
Head and neck squamous cell carcinoma	ARPC5	Inhibition	^[13,44,45]^
Colorectal cancer	Arp2, Arp3	Inhibition	^[34,53,54]^
Pancreatic cancer	ARPC3, ARPC4	Significant inhibition	^[47–49]^
Gastric cancer	Arp2, Arp3, ARPC2	Inhibition	^[51,52]^
Breast cancer	Arp2 (Arp2 is co-expressed with WAVE2)	Significant inhibition	^[55–57]^
Hepatocellular carcinoma	ACTR2, ACTR3, ARPC1A, ARPC1B, ARPC2	Inhibition	^[59]^
Lung squamous cell carcinoma	ARPC5	Significant inhibition	^[58]^

Arp2/3 = actin-related protein 2/3, WAVE = WASP famly verprolin-homologous protein family

### 6.1. The Arp2/3 complex in head and neck squamous cell carcinoma (HNSCC)

Worldwide, head and neck cancer is the sixth most common malignancy in the world, with an annual incidence of 830,000 new cases and around 430,000 deaths each year.^[44]^ Kinoshita et al^[45]^ discovered that the ARP2/3 complex’s subunit 5 (ARPC5) was expressed at significantly higher levels in HNSCC tissues than in normal tissues, contributing to cancer cell migration and invasion. They also suggested that the ARPC5 gene was directly regulated by miR-133a and that restoring miR-133a and silencing ARPC5 resulted in reorganization of the actin cytoskeleton and changes in cell morphology to a rounded, vesicular shape, ultimately significantly inhibiting the migration and invasion of cancer cells. Cortactin is an actin 2/3 (Arp2/3) complex activating and filamentous (F) actin-binding protein that is associated with tumor cell invasion and migration.^[45]^ According to Rothschild et al,^[13]^ HNSCC cells with cortactin overexpression show higher Arp2/3 complex activity and are more aggressive and migratory than HNSCC cells without cortactin overexpression. In conclusion, the Arp2/3 complex plays an important role in the development of head and neck squamous cell carcinoma and may be a potential therapeutic target.

### 6.2. The Arp2/3 complex in pancreatic cancer

Pancreatic cancer (PC) is the most lethal neoplastic epithelial tumor, with a 5-year overall survival rate of about 10%, and is anticipated to overtake lung cancer as the second biggest cause of cancer-related deaths by 2024.^[46]^ Rauhala et al^[47]^ discovered that ARPC3 and ARPC4 were the most highly expressed subunits of the Arp2/3 complex in pancreatic cancer and that silencing of Arp complex subunits, particularly ARPC4, significantly reduced pancreatic cancer cell migration, implying that the Arp2/3 complex plays a key role in pancreatic cancer cell invasion and migration. In another study, Zhao et al^[48]^ demonstrated that *mTOR* (mechanistic target of rapamycin kinase) complex 1 (*mTORC1*) and 2 (*mTORC2*) perform dual but non-duplicative regulatory functions in acinar-to-ductal metaplasia (ADM) and early pancreatic carcinogenesis by boosting Arp2/3 complex function and causing actin cytoskeleton remodeling.

Latario et al^[49]^ found and defined TMTs linked with pancreatic cancer treatment resistance in the Dartmouth-Hitchcock pancreatic cancer (DHPC)-018 pancreatic cancer cell line, concluding that TMT assembly required actin polymerization of the Arp2/3 complex.

### 6.3. The Arp2/3 complex in gastric cancer

Gastric cancer is one of the most prevalent tumors of the digestive system, with the fifth highest incidence (5.6%) and fourth highest death rate (7.7%) among cancers globally, posing a serious threat to human health.^[50]^ In a study on gastric cancer, Zheng et al^[51]^ found that the expression of Arp2 and Arp3 proteins were both significantly higher in gastric cancer than in gastritis by immunohistochemistry and further suggested that there was a positive correlation between the expression of Arp2 and Arp3 proteins and the size, depth of infiltration, and venous infiltration of tumor tissue. According to Zhang et al,^[52]^ ARPC2 expression was reported to be higher in gastric cancer tissues than in normal gastric tissues and was associated with a high tumor stage, lymph node infiltration, and poor prognosis in patients.

### 6.4. The Arp2/3 complex in colorectal cancer

Colorectal cancer has become the third leading cause of cancer-related deaths in humans, accounting for 8.7% of all cancer deaths.^[1]^ By immunohistochemistry, Otsubo et al^[53]^ discovered significant overexpression of Arp2 and Arp3 proteins in cancer cells and stromal cells in 175 colorectal cancer specimens, whereas Arp2 and Arp3 were not expressed in normal colorectal mucosal epithelial or stromal cells. They proposed that stromal cells expressing Arp2 and Arp3 create a microenvironment that promotes cancer cell invasion prior to invasion. According to Yan et al,^[34]^ *LASP1* interacts with N-WASP via the SH3 domain, promoting N-WASP binding and activating the Arp2/3 complex and actin polymerization, thus contributing as a guide to colorectal cancer invasion and migration. In another study, Rada et al^[54]^ discovered that Runt-related transcription factor-1 (*RUNX1*) was overexpressed in vascular-selective colorectal cancer liver metastasis (CRCLM) tumor cells and that being an upstream positive regulator of the Arp2/3 complex expression level could drive cancer cell motility through the Arp2/3 complex to achieve vascular coexistence, implying that ARP2/3 is a key mediator of vascular coexistence in CRCLM14. In conclusion, aberrant expression and activation of the Arp2/3 complex contribute to the invasion and metastasis of colorectal cancer.

### 6.5. The Arp2/3 complex in breast cancer

Breast cancer is now the most prevalent malignancy among women and has become one of the leading causes of cancer-related deaths in the female population due to its aggressive, metastatic, and treatment-resistant nature. Excessive protrusions in breast cancer cells are one of the reasons why breast cancer has more aggressive and metastatic properties. In a study of invasive breast cancer, Iwaya et al^[55]^ proposed that Arp2 is co-expressed with WAVE2 and that the Arp2/3 complex drives actin polymerization to form lamellipodial protrusions upon activation by WAVE2. Further research discovered that *HER2* gene amplification might govern the generation of invasive pseudopods by activating the WAVE2-Arp2/3 signaling pathway, which in turn increased MMP-independent breast cancer cell invasion and migration.^[56]^ In another study, Ko et al^[57]^ found that inhibiting the Rac1/WAVE2/Arp2/3 pathway with pterostilbene greatly reduced the invasion and migration of MDAMB-231 breast cancer cells without causing cytotoxicity. In conclusion, the Arp2/3 complex plays a key role in the invasion and migration of breast cancer by mediating actin polymerization.

### 6.6. The Arp2/3 complex in other cancers

In their study of the regulatory mechanisms of lung squamous cell carcinoma development, Moriya et al^[58]^ found that the ARPC5 protein was highly expressed in lung squamous cell carcinoma compared with normal lung tissue and that the invasive and migratory abilities of PC10 cells could be significantly inhibited by downregulating ARPC5 gene expression in the cell line PC10. Through various bioinformatics analysis methods, Huang et al^[59]^ discovered that upregulation of Arp2/3 complex expression in hepatocellular carcinoma tissues was closely associated with poorer overall survival in hepatocellular carcinoma patients, and further suggested that the Arp2/3 complex’s Arp2, ARPC2, and ARPC5 subunits could be independent risk factors for survival in hepatocellular carcinoma patients. Kumagai et al^[60]^ used gene microarray analyses and other methods to find that the ARPC1B subunit of the Arp2/3 complex could be used as a predictive marker for the sensitivity of choroidal malignant melanoma to radiation therapy, and that the mechanism may be related to the regulation of actin polymerization by the Arp2/3 complex. In conclusion, the Arp2/3 complex is closely associated with the invasion and migration of these cancers due to its function in mediating actin polymerization, and whether the Arp2/3 complex plays a similar role in other types of cancer needs to be further investigated.

## 7. Possible targets for drug therapies for preventing metastasis and invasion

In the last decade, we have made significant progress in understanding the role of the Arp2/3 complex in cancer research. However, there are still certain connections in the signaling cascade of the Arp2/3 complex that remain to be explored, and they may eventually become targets for potential anticancer drugs. In one study, by blocking the binding of ARPC2 to ventolin, Pimozide, an ARPC2 inhibitor, decreased lamellipodia expansion and cell spreading, and hence cancer cell migration.^[61]^ In another study on small molecule inhibitors of ARPC2, benproperine could inhibit the function of the Arp2/3 complex by binding directly to the ARPC2 subunit, leading to inhibition of actin polymerization and disruption of lamellar structures, ultimately inhibiting cancer cell migration and metastasis without inhibiting normal cells.^[61]^ So far, ARPC2 is a promising target for anti-cancer therapy, even though no anti-cancer drug targeting ARPC2 has been used in the clinic yet. The Arp2/3 complex consists of seven subunits, some of which have different isoforms, and the expression of each subunit may vary between different cancer cells. Therefore, other subunits of the Arp2/3 complex and their isoforms may also be targets for anti-cancer therapy.

## 8. Discussion

Cancer cell invasion and metastasis is a major cause of cancer death and a complex multi-step process that involves the remodeling of the actin cytoskeleton and the formation of cellular pseudopods. Arp2/3 complex activity has been shown to correlate with the aggressiveness and metastatic potential of cancer cells. Given the close relationship between the Arp2/3 complex and cancer metastasis, the regulation of the nucleation activity of the Arp2/3 complex has become the focus of anti-cancer metastasis therapy. Although some small molecule inhibitors of the Arp2/3 complex inhibit metastasis by targeting Arp2 and Arp3 to inhibit actin filament nucleation, unfortunately, these inhibitors also inhibit the migration of normal cells. The Arp2/3 complex consists of seven subunits, some of which have different isomers, such as the Arp3, ARPC1, and ARPC5 subunits. Thus, the discovery of differential expression of the Arp2/3 complex in normal versus cancerous tissues may provide a direction for addressing the above questions. Benproperine selectively inhibits the migration and invasion of cancer cells by targeting and inhibiting the function of the ARPC2 subunit, and, interestingly, Benproperine does not affect the expression levels of the Arp2/3 complex subunit or the migration of normal cells.^[61,62]^ Thus, small molecule inhibitors that target the Arp2/3 complex subunit and impair its function may be a new avenue for cancer therapy, and evidence suggests that ARPC2 is also a promising potential target for cancer diagnosis and treatment.

Activation of the Arp2/3 complex is regulated by multiple phospho-modification, and multiple regulator, including PCK1, PIK4, and NIK, which can phosphorylate the Arp2/3 complex, are potential targets against cancer invasion. The function of the Arp2/3 complex in mediating actin polymerization requires not only its own phosphorylation modification but also activation or promotion by various nucleation-promoting factors, such as cortactin, WASP (Wiskott-Aldrich syndrome protein) family proteins, WAVE (The WASP family verproline-homologous protein), etc., all of which may be potential targets for anti-invasive cancer therapy. Cortactin, N-WASP, and WAVE all bind and activate the Arp2/3 complex with specific domains and also have numerous phosphorylation sites, which in turn influence their ability to bind and activate the Arp2/3 complex. Thus, these phosphorylation modifications of NPF and regulatory factors that can phosphorylate NPF, such as *Shp1, Dyrk1A*, Casein Kinase II, *PAK4, SRC, ERK, Abl, Cdk5*, etc., may provide targets for anti-invasive cancer therapy. In conclusion, how to reduce the expression and activity of the Arp2/3 complex in cancer tissues is the central issue in formulating cancer anti-invasive therapy around the Arp2/3 complex.

## Author contributions

**Project administration:** Sen Li, Wenjian Hu.

**Writing – original draft:** Fengfeng Qin, Ji Yin, Daiying Li, Yanlin Huang, Lanxin Hu, Lu He, Caifeng Lv, Xiaohui Li.

**Writing – review & editing:** Sihan Zheng.
